# Treatment duration and factors associated with the surgery-first approach: a two-center study

**DOI:** 10.1186/s40510-015-0101-1

**Published:** 2015-09-10

**Authors:** Flavio Uribe, Sara Adabi, Nandakumar Janakiraman, Veerasathpurush Allareddy, Derek Steinbacher, David Shafer, Carlos Villegas

**Affiliations:** Division of Orthodontics, Department of Craniofacial Sciences, University of Connecticut School of Dental Medicine, 263 Farmington Avenue, Farmington, CT 06030 USA; Division of Orthodontics, University of California at San Francisco, San Francisco, CA 94143 USA; Department of Orthodontics, College of Dentistry, The University of Iowa, Iowa City, IA USA; Department of Pediatrics, Dental Services, Oral Maxillofacial and Craniofacial Surgery, Yale School of Medicine, New Haven, CT USA; Division of Oral and Maxillofacial Surgery, Department of Craniofacial Sciences, University of Connecticut School of Dental Medicine, Farmington, CT USA; Department of Orthodontics and Maxillofacial Surgery, CES University and Private Practice Medellin, Medellin, Colombia

## Abstract

**Background:**

The aim of this study was to evaluate treatment duration and number of appointments in orthognathic surgery using the surgery-first approach (SFA) and to evaluate the factors associated to these outcomes.

**Methods:**

This was a retrospective chart review of consecutively treated patients with SFA at a University clinic and a private practice setting. Treatment duration, number of appointment, and factors associated to this duration such as patient demographics, type of surgery, use of 3D planning, and treatment center among others were evaluated. Multivariable linear regression models were used to examine the simultaneous association between all predictor variables and outcomes.

**Results:**

Median treatment duration for patients undergoing SFA was 9.6 months [6.1 (25 % percentile); 13.4 (75 % percentile)] with a median number of 13.8 appointments [9 (25 % percentile); 17 (75 % percentile)]. Transverse maxillary expansion was associated with longer treatment duration and number of appointments. There was also a significant difference in number of appointments between the two treatment centers.

**Conclusions:**

SFA significantly reduces treatment duration in orthognathic surgery. Transverse expansion is associated with longer treatment duration and number of appointments.

## Background

Conventional orthognathic surgery with its three distinct phases (pre-surgical, surgical, post-surgical) appears to entail prolonged treatment times. Few studies have examined treatment duration for surgical orthodontic treatment with some of the studies having separately analyzed the treatment duration of each phase. Proffit and Miguel [1] reported median duration of orthodontic treatment ranging from 18–28 months depending on whether the treatment was carried out in a faculty practice, a university clinic, or outside the university. Dowling et al. [2] concluded that the median time to complete treatment was 21.9 months, with median treatment times of 15.4 and 5.9 months for pre-surgical and post-surgical phases, respectively. Another study based in the UK [3, 4] also reported very similar treatment duration times for both the pre-surgical and post-surgical phases. Slavnic and Marcusson [5] in a recent study on combined orthodontic-orthognathic treatment reported total orthodontic treatment time of 27.8 months with pre-operative orthodontic treatment time of 16.7 months and post-operative treatment time of 4.6 months in patients treated in a university hospital orthodontic clinic. Pre-surgical orthodontics is indeed the longest phase. This was validated by O’Brien et al. [6] in a prospective multi-center study which found that the total combined treatment takes 32 months on average, with a pre-surgical orthodontic phase lasting approximately 25 months. Similarly, Diaz et al. [7] found a pre-surgical treatment time in orthodontics of 24 months followed by 12 months of post-surgical orthodontics in a Caucasian population.

Surgery-first approach (SFA) is a term applied to the approach in orthognathic surgery where the pre-surgical orthodontic phase is totally obviated. The surgical procedure is conducted without any alignment or leveling of the arches, and orthodontic treatment is initiated a short period after the osteotomies. Liou et al. [8] described the guidelines for candidates to receive this type of approach. Almost every type of malocclusion and dentofacial deformity has been reported to be amenable to treatment with SFA. Published reports include surgery first for class II, class III, deep bites, open bites, and asymmetries. However, the majority of the published cases involve patients with class III malocclusions [9–11].

Some of the advantages that are claimed with this approach are the immediate resolution of dentofacial deformity, the easier decompensation of the malocclusion after surgery by eliminating the soft tissue resistance to orthodontic tooth movement, and most important of all, the significant reduction in treatment time.

The reduction in treatment time has been one of the most important characteristics that have made this approach appealing to patients, surgeons, and orthodontists. The specific duration of treatment for patients that have received SFA has been reported in the literature mostly through isolated case reports. Most of these report excellent treatment results achieved in less than 1 year of total treatment time. A retrospective study evaluating class III patients treated with this approach, reported approximately 18 months of treatment time that included a short orthodontic preparation time of 3 weeks prior to surgery [12]. Recently, a systematic review [13] reported mostly on cases reports or case series and found that treatment duration was approximately less than a year.

The aim of this study is to report on a series of consecutive patients treated in two different centers by two different orthodontists with the SFA. The primary outcome was to evaluate the length of treatment and number of appointments of patients undergoing SFA. Secondary outcome was to evaluate any association between the duration of treatment and number of appointments with patient’s demographics, type of dentofacial deformity, type of surgery, use of virtual 3D planning, and treatment center among other variables.

## Methods

This retrospective cohort two-center study evaluated patients that were treated with the SFA during a 7-year period (2008–2015). All subjects had undergone orthognathic surgery with SFA. Surgeries were performed by three different experienced surgeons in three different locations (Yale, New Haven; University of Connecticut; and Private Practice Dr. Carlos Villegas in Medellin, Colombia). Two different orthodontists in two centers were involved in the treatment (Private Practice in Medellin, Colombia, and at the University of Connecticut). In one of the centers (Colombia), the oral surgeon was dual trained and performed both treatments. The two centers pertained to the location of the orthodontist, and thus, one was in Medellin, Colombia, and the other at the University of Connecticut.

All patients that had undergone a surgery-first approach and had complete hard copy and digital records were included. Exclusion criteria were patients with syndromes or craniofacial deformities such as cleft lip and palate or any patient that had a phase of pre-surgical orthodontics. Also, any patient that had an unfavorable fracture, which required a second surgery at a later timepoint was excluded. This study was approved by the institutional review board for human subjects research at the University of Connecticut (IRB# 15-145-2).

### Protocol for surgery first

Records consisting of photographs, dental models, and radiographs (in some instances cone-beam computed tomographs (CBCTs)) were taken and submitted for insurance approval for those patients that had coverage. Virtual 3D planning with the fabrication of a splint using CAD/CAM technology was used in some patients, specifically those who had a noticeable asymmetry in the clinical exam. However, the majority of patients had conventional model surgery planned in an articulator. Patients were bonded with orthodontic appliances 1 to 21 days prior to the surgical intervention. No wire or an orthodontic wire (0.016-in., 0.016 × 0.016-in., or 0.016 × 0.022-in. NiTi) was placed either the day before surgery or at the time of surgery. In one patient, a customized passive of 0.017 × 0.025 NiTi archwire was placed in both arches prior to surgery.

Surgical procedure was conducted as planned using the splints as guides for the final position of the jaws. Splints were removed at the time of surgery for all patients except for those where maxillary transverse expansion was performed. In these patients, the splint was left in place from 4–6 weeks. Patients wore intermaxillary elastics from the day of surgery through the post-surgical orthodontic phase as needed.

Orthodontic treatment was resumed from 2–6 weeks after the surgical procedure by removing the surgical archwires and placing NiTi wires with intermaxillary elastics for the initial aligning and leveling phase. Patients were seen every 2–4 weeks for follow-ups that included wire changes and an alteration in the force vector of the elastics when required. Few patients did not follow this visit schedule and had more spaced appointments. Patients were debonded when a good occlusal outcome was achieved. This was determined by the treating orthodontist. In some few patients, appliances were removed slightly early as patients were satisfied with the facial and dental esthetic outcome and requested to finalize the orthodontic treatment. At the time of appliance removal, patients were given retainers for either full-time or night-time wear depending on the orthodontist.

### Statistical analysis

Simple descriptive statistics were used to summarize the data. The outcome variables of interest included the total treatment duration and total number of visits. Both these variables were used as continuous variables. The predictor variables of interest included the following: age, gender, type of malocclusion, type of surgery, vertical pattern, need for expansion due to transverse deficiency, presence of significant amount of asymmetry, use of 3D planning, use of miniplates or mini-implants over the course of orthodontic treatment, performance of genioplasty, and treatment center (USA versus Colombia). Age was used as a continuous variable while all other predictor variables were used as categorical variables. Two multivariable linear regression models (separate models for each outcome) were used to examine the simultaneous association between all predictor variables and outcomes). Multivariable regression model was fit using the ordinary least squares approach. For each level of predictor variables, the estimated change in outcome variable and the associated 95 % confidence intervals (CI) were computed. For the two multivariable linear regression models, the total variance accounted by all predictor variables were computed. All statistical tests of association were two-sided and a *p* value of <0.05 was deemed to be statistically significant. All statistical analyses were performed using SPSS Version 22.0 for Windows software (IBM Corp, New York).

## Results

Data for a total of 66 subjects (47 from Colombia and 19 from USA) was collected in this study. Only two patients of those that underwent surgery first in this two-center study were excluded. The reason for exclusion for one of them was an unfavorable fracture of the mandible that required a second surgery 6 months after healing. The second patient was excluded since the hard copy chart was not found. Characteristics of the study subjects are summarized in Table 1. The mean age was 23.8 years (median was 21 years). Females comprised majority of the subjects (68.2 %). Most of the malocclusions were class III (77.3 %). Two-jaw surgery was performed in 62.1 % of the subjects. Majority of the patients had a normofacial (66.7 %) vertical pattern. A vast majority (90.9 %) of subjects did not require expansion for transverse deficiencies. One patient required constriction of the maxilla with surgery. Close to 38 % had a significant asymmetry. 3D planning was used for 24.2 % of subjects. Miniplates or mini-implants were used over the course of orthodontic treatment in 25.8 % of subjects. About 44 % also had a genioplasty with the orthognathic surgery. Five patients received fat grafting of the lips, cheeks, etc. at the time of surgery. Other ancillary procedures included the following: condylectomy (four patients), mandibular basal ostectomy (three patients), and submental lipectomy (one patient). Only one patient had an extraction of a maxillary premolar that was fully blocked out of the arch. Conventional orthodontic appliances were placed in all patients, except for three patients that had customized appliances. These customized appliances consisted of customized labial archwires (Suresmile, OraMetrix, Richardson, TX) for one patient and lingual appliances (Harmony, American Orthodontics, Sheboygan, WI) for another. A third patient received an indirect bonding setup based on a virtual orthodontic plan (Orthocad, Cadent Inc, Carlstadt, NJ). The mean treatment time was 10.5 months (median = 9.6 months). The mean number of visits was 13.8 per subject (median = 13 visits).

**Table 1:** Characteristics of patients

Characteristic		*N* = 66
Age	Mean	23.8 years
	Median	21 years
Gender	Female	68.2 %
	Male	21 %
Malocclusion	Class I	13.6 %
	Class II	9.1 %
	Class III	77.3 %
Type of surgery	One jaw	37.9 %
	Two jaw	62.1 %
Vertical pattern	Normofacial	66.7 %
	Dolicofacial	30.3 %
	Brachifacial	3 %
Transverse	Expansion	9.1 %
	No expansion	90.9 %
Presence of significant asymmetry	No	62.1 %
	Yes	37.9 %
Use of 3D plan	No	75.8 %
	Yes	24.2 %
Use of mini plates or mini-implants	No	74.2 %
	Yes	25.8 %
Performance of genioplasty	No	56.1 %
	Yes	43.9 %
Center	Colombia	71.2 %
	USA	28.8 %
Total treatment time	Mean	10.5
	Minimum	2.2
	25th percentile	6.1
	Median	9.6
	75th percentile	13.4
	Maximum	34.8
Number of visits	Mean	13.8
	Minimum	2
	25th percentile	9
	Median	13
	75th percentile	17
	Maximum	43

Summary of estimates from multivariable linear regression models examining the simultaneous association of all predictor variables and outcomes (treatment time and number of visits) are summarized in Table 2. Following adjustment of all predictor variables, those with class II malocclusions were associated with short duration of treatment time (estimate is −5.83, *p* = 0.02) when compared to those with class III malocclusions, and those that required transverse expansion were associated with longer duration of treatment (estimate is 10.21, *p* = 0.001). None of the other predictor variables were significantly associated with total treatment time in the multivariable linear regression model. This multivariable regression model accounted for 46.3 % of variance in the total treatment time. Following adjustment of all predictor variables, those that required transverse expansion were associated with significantly more number of visits (estimate is 8.64, *p* = 0.009) and those that were treated in USA were also associated with significantly more number of visits (estimate is 6.36, *p* = 0.03). None of the other predictor variables were significantly associated with total number of visits in the multivariable linear regression model. This multivariable regression model accounted for 54.2 % of variance in the total number of visits.

**Table 2:** Summary of estimates from multivariable linear regression models examining the simultaneous association between predictor variables and outcomes

Predictor variables	Outcome is treatment time		Outcome is number of visits	
	Estimate (95 % CI)	*p* value	Estimate (95 % CI)	*p* value
Age (each 1 unit increase)	0.02 (−0.14–0.19)	0.78	0.14 (−0.04–0.32)	0.12
Gender		0.22		0.65
Male	1.86 (−1.14–4.86)		0.72 (−2.49–3.92)	
Female	Reference		Reference	
Type of malocclusion				
Class I	1.59 (−2.62–5.80)	0.45	−1.38 (−5.87–3.12)	0.54
Class II	−5.83 (−10.92 to −0.75)	0.02	−4.55 (−9.98–0.87)	0.10
Class III	Reference		Reference	
Vertical pattern				
Dolicofacial	−2.95 (−6.35–0.44)	0.44	−1.46 (−5.08–2.16)	0.42
Brachifacial	6.53 (−1.26–14.32)	14.32	5.37 (−2.95–13.69)	0.20
Normofacial	Reference		Reference	
Transverse expansion		0.001		0.009
Yes	10.21 (4.26–16.17)		8.64 (2.29–15.00)	
No	Reference		Reference	
Significant asymmetry		0.99		0.55
Yes	0.01 (−3.28–3.30)		−1.04 (−4.55–2.47)	
No	Reference		Reference	
3D Plan		0.12		0.07
Yes	−3.44 (−7.77–0.90)		−4.25 (−8.87–0.38)	
No	Reference		Reference	
Type of surgery		0.47		0.92
Two jaws	−1.10 (−4.14–1.93)		0.16 (−3.08–3.40)	
One jaw	Reference		Reference	
Use of TADs		0.65		0.33
Yes	0.83 (−2.82–4.48)		1.90 (−2.00–5.80)	
No	Reference		Reference	
Genioplasty		0.79		0.83
Yes	0.41 (−2.75–3.58)		−0.35 (−3.73–3.02)	
No	Reference		Reference	
Center		0.31		0.03
USA	1.95 (−1.85–5.74)		6.36 (2.31–10.41)	
Colombia	Reference		Reference	

## Discussion

This is the first study to evaluate treatment duration, number of appointments, and factors associated to these outcomes with SFA in two centers. One of the most highlighted benefits of SFA is the reduction in treatment duration. Recently, a systematic review evaluated a few retrospective cohort studies and a larger number of case reports treated with SFA [13]. The results showed that the majority of cases were treated under a year. This agrees with our findings which show an average treatment duration of 10.5 months. This is a clear advantage over the conventional approach where treatment times have been reported in the realm of 18–36 months [1, 5–7]. It should be noted that the number of appointments is slightly higher than in the conventional approach where patients are seen on a once-a-month visit schedule. This is because the patients are scheduled on a shorter appointment interval schedule (usually 2–3 weeks). Our average number of appointment is however far less than the 22 orthodontic appointments reported by a recent study evaluating 45 consecutive patients treated with SFA [14].

It should be noted there was a wide range in the treatment duration and number of appointments. Those patients with long treatment times were associated to failed visits or complications after surgery where the planned outcome was not achieved.

We found that the transverse dimension had a significant influence in treatment duration and number of appointments in the multivariate regression models. It is likely that these patients had more complex surgeries where more segments needed to be controlled. Since the transverse dimension also tends to relapse the most, the extended treatment times could be related to the orthodontic treatment trying to control for these relapse tendencies. Another contributing factor could have been that the segmentation of the maxilla precluded from adequate occlusal outcomes after surgery, which resulted in more complicated post-surgical orthodontics. Finally, it should be noted that patients that received transverse expansion during surgery required a 4–6 weeks waiting period before removing the splint prior to starting orthodontic treatment.

We did not find that there was difference in results in the treatment duration between surgeons and orthodontists at the two centers. However, there was a difference in number of appointments, with the patients in the US having more appointments. This finding suggests that patients were seen more often in the US since treatment duration was not different between the centers. Since all the surgeons and orthodontists in both centers were experienced, it was not surprising that treatment duration was not different.

Interestingly, class II patients were associated with shorter treatment times. This could be associated to possible less dental compensations in these type of malocclusions, which would result in less treatment times. However, it should be noted that the number of patients with class II occlusion was minimal compared to the class III patients; therefore, this finding needs to be evaluated further with a larger sample size.

CAD/CAM technology has become popular in orthognathic surgery as it is believed that treatment planning can be facilitated and more accurate outcomes can be achieved [15]. When evaluating this factor in relation to treatment duration and number of appointments, we did not find a significant association. It should be noted that this approach was reserved primarily for patients with significant asymmetries. It is possible that the treatment time could have extended further if not approached with this type of planning.

The limitations of the study are those known to retrospective studies. There may be a bias from the clinicians to end these treatments faster. Additionally, this study, as many others, did not objectively quantify the quality of orthodontic outcome. Although patient satisfaction is high as reported by Hernandez-Alfaro et al. [14], it has been noted by the orthodontists treating some of these patients that there is an urge to remove the orthodontic appliances very soon after orthognathic surgery.

There is clear interest in accelerating tooth movement and reducing treatment times in conventional and surgical orthodontics [16–18]. SFA appears to be a promising approach to achieve this goal in orthognathic surgery.

## Conclusions

This study reported on a retrospective two-center study evaluating treatment duration and number of appointment in patients treated with SFA and factors associated to these two outcomes. It appears that SFA significantly reduces treatment time as observed in both centers. Expansion of the transverse dimension in surgery first is associated with longer treatment times and number of appointments.
